# Structural Barriers Underlying Kidney and Liver Transplant Inequity in Hispanic Americans

**DOI:** 10.7759/cureus.117431

**Published:** 2026-10-04

**Authors:** Aarnav Gorantla, Emily Herrington, Giuseppe Orlando, Girish Mour

**Affiliations:** 1 Health Equity Research, Wake Forest Institute for Regenerative Medicine, Durham, USA; 2 College of Humanities and Social Sciences, North Carolina State University, Chapel Hill, USA; 3 Transplant Surgery, Advocate Health Care, Atrium Wake Forest Baptist Medical Center, Winston Salem, USA; 4 Transplant Nephrology, Mayo Clinic in Arizona, Phoenix, USA

**Keywords:** health equity, hispanic americans, hispanic paradox, kidney transplantation, liver transplantation, organ transplantation, structural barriers, transplant disparities

## Abstract

Hispanic patients in the United States experience a disproportionate burden of end-stage kidney and liver disease, yet they continue to encounter disparities across multiple stages of the transplantation pathway. This narrative review synthesizes evidence on transplant referral, evaluation, waitlisting, organ allocation, living and deceased donation, structural barriers, interventions, and posttransplant outcomes among Hispanic patients. National and regional studies demonstrate persistent inequities whose magnitude varies by organ, stage of care, geographic region, and policy era, with reduced access to both deceased-donor and living-donor kidney transplantation reported among Hispanic adults and children relative to their White counterparts. Linguistic isolation, geographic variation in organ availability and transplant infrastructure, socioeconomic disadvantage, insurance limitations, medical distrust, and organ-procurement practices may contribute to these disparities. Culturally and linguistically concordant programs have improved transplant knowledge, waitlist access, and living-donor transplantation in selected settings. Despite reduced access, Hispanic kidney and liver transplant recipients frequently demonstrate outcomes comparable with or better than non-Hispanic White recipients, although the mechanisms underlying this pattern remain uncertain. Because several major studies use overlapping national registry populations, their findings are best interpreted as convergent rather than fully independent evidence. Structural interventions across referral, evaluation, language access, geography, financial support, organ procurement, and transplant-center practice may improve transplantation equity.

## Introduction and background

Hispanic individuals constitute the largest racial or ethnic minority population in the United States and represent a growing proportion of the population [1]. This demographic trajectory has important implications for transplantation because Hispanic patients experience a substantial burden of chronic diseases associated with organ failure, particularly type 2 diabetes mellitus, hypertension, obesity, and chronic kidney disease [2]. Hispanic populations also experience a high burden of metabolic dysfunction-associated steatotic liver disease (MASLD) and related progression to metabolic dysfunction-associated steatohepatitis (MASH), cirrhosis, and hepatocellular carcinoma (HCC), with food insecurity, limited healthcare access, language barriers, and health-literacy challenges further influencing disease burden and access to specialty care [3,4].

Transplantation for kidney and liver failure follows a sequential pathway that includes specialty referral, pretransplant evaluation, waitlisting, organ allocation, living or deceased donation, transplantation itself, and posttransplant follow-up care; disparities can arise at any of these stages rather than at a single point of access.

National Organ Procurement and Transplantation Network/Scientific Registry of Transplant Recipients (OPTN/SRTR) data show that Hispanic patients account for a substantial share of kidney and liver transplant candidates, comprising 22.3% of adults on the kidney waiting list and 19.9% of adults on the liver waiting list at the end of 2024. However, they contribute to a comparatively smaller share of the donor pool, at 16.3% of living kidney donors and 14.6% of deceased liver donors that same year [5]. This asymmetry between candidate-side and donor-side representation is a population-level context rather than direct proof of inequity, because disease prevalence, eligibility, organ type, candidate characteristics, and allocation rules differ across these denominators. Stronger evidence comes from stage-specific cohort and registry studies examining referral, evaluation, waitlist progression, living donation, deceased-donor transplantation, waitlist mortality, and posttransplant outcomes.

An important counterpoint is that Hispanic patients who undergo kidney or liver transplantation frequently demonstrate post-transplant outcomes comparable with or better than those of non-Hispanic White recipients [6-8]. This pattern has been described as a transplant-specific manifestation of the broader Hispanic paradox [9]. Several mechanisms may contribute, including selection into transplantation, differences in age and comorbidity, social and family support, center-level effects, adherence, socioeconomic factors incompletely captured in registry data, and other biological or behavioral influences. Available observational studies cannot determine their relative contributions.

Although Hispanic transplantation inequity has been examined across individual organs and stages of care, recent narrative work has examined Hispanic disparities within kidney disease and transplantation specifically [10]. This review extends that perspective by examining kidney and liver transplantation together as an interconnected pathway, focusing on where disparities emerge, which structural mechanisms recur across studies, whether culturally and linguistically concordant interventions can improve access, and which system-level responses are most directly supported by the available evidence.

## Review

A targeted literature search was conducted to identify studies from USA addressing transplantation access, waitlisting, organ allocation, living and deceased donation, posttransplant outcomes, structural barriers, and disparity-reduction interventions among Hispanic patients. PubMed/MEDLINE was searched for English-language literature published from January 2000 through May 2026 using combinations of terms related to Hispanic or Latino ethnicity, kidney and liver transplantation, living and deceased donation, waitlisting, transplant disparities, language, geography, socioeconomic determinants, and organ procurement. Additional relevant publications were identified through targeted searches of literature indexed in Embase, the Cumulative Index to Nursing and Allied Health Literature (CINAHL), and Scopus, and through reference lists of key articles.

A representative search strategy combined terms such as (Hispanic OR Latino) AND (kidney transplant* OR liver transplant* OR renal transplant*) AND (disparit* OR inequit* OR access OR waitlist* OR allocation OR donation), with additional terms for language, geography, socioeconomic status, and organ procurement, adapted as appropriate for each database. Candidate studies identified through these searches and reference-list screening were reviewed for relevance to Hispanic or Latino kidney or liver transplant access, structural barriers, interventions, or post-transplant outcomes and incorporated when they provided US-based, ethnicity-stratified data or directly relevant contextual or policy information. Consistent with the narrative-review design, formal duplicate screening, adjudication, and risk-of-bias scoring were not performed; interpretation instead emphasized study population, design, data source, era, comparator, adjustment strategy, and relevance to the transplantation stage. Studies were selected to represent major stages of the transplantation continuum.

Organ Procurement and Transplantation Network/Scientific Registry of Transplant Recipients (OPTN/SRTR) and United States Renal Data System (USRDS) reports were used for population-level and policy context. As this was a narrative rather than a systematic review, the search was not designed to provide exhaustive coverage through fully reproducible multi-database methods, and formal Preferred Reporting Items for Systematic Reviews and Meta-Analyses (PRISMA) study accounting was not performed. Quantitative findings are reported in the metric used by the original investigators and are not pooled. Attention was given to population, transplant stage, data source, study era, comparator, and adjustment strategy. As several publications use overlapping USRDS, OPTN, and SRTR populations, repeated registry findings are interpreted as convergent evidence rather than as fully independent replications. The literature search and evidence synthesis strategy is summarized in Figure 1, and representative quantitative findings across the transplantation pathway are summarized in Table 1. 

**Figure 1:**
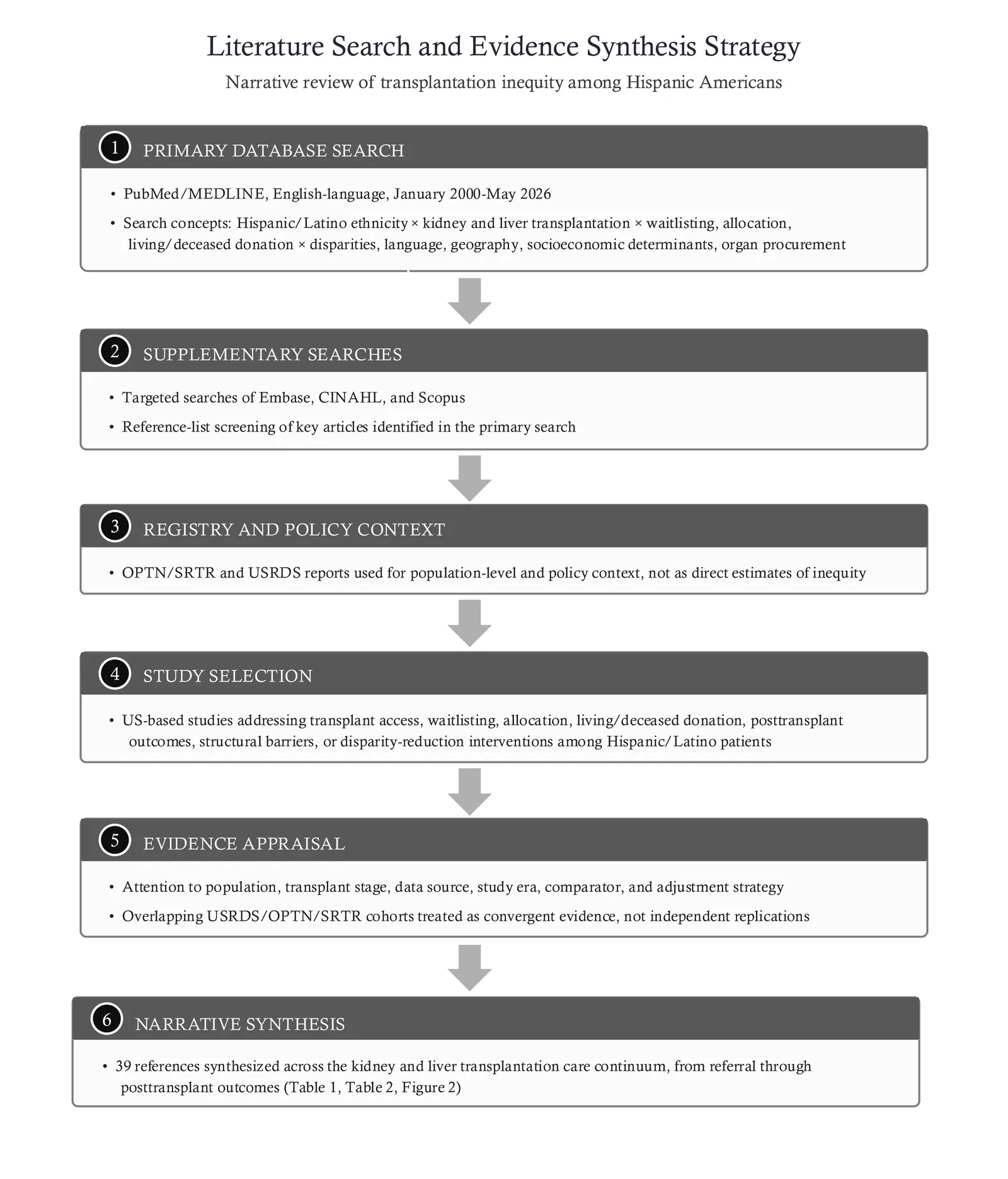
Literature search and evidence synthesis framework for this narrative review. Search concepts combined Hispanic/Latino ethnicity with kidney and liver transplantation across referral, waitlisting, allocation, and donation, supplemented by targeted searches of additional databases, reference-list screening, and national registry reports for population-level context. As this is a narrative rather than systematic review, the search was not designed for exhaustive or fully reproducible multi-database retrieval, and formal PRISMA study accounting was not performed. CINAHL: Cumulative Index to Nursing and Allied Health Literature; OPTN: Organ Procurement and Transplantation Network; SRTR: Scientific Registry of Transplant Recipients; USRDS: United States Renal Data System.

**Table 1:** Representative quantitative evidence across the transplantation pathway. Representative quantitative findings on Hispanic-White or Hispanic-Latino disparities across sequential stages of kidney and liver transplantation, drawn from national registry, cohort, and program-evaluation studies. Estimates are reported in the metric used by the original investigators and are not pooled. aHR: adjusted hazard ratio; aOR: adjusted odds ratio; CI: confidence interval; DDKT: deceased-donor kidney transplantation; HKTP: Hispanic Kidney Transplant Program; HR: hazard ratio; KAS: Kidney Allocation System; LDKT: living-donor kidney transplantation; NASH: nonalcoholic steatohepatitis; OPO: organ procurement organization; OR: odds ratio; RR: risk ratio; SHR: subdistribution hazard ratio; UNOS: United Network for Organ Sharing; USRDS: United States Renal Data System.

Stage	Study	Population/source	Key estimate	Interpretive boundary
Predialysis care	Purnell et al. [11]	USRDS adults initiating dialysis	aOR 0.61	Predialysis care, not direct transplant referral
Pediatric preemptive LDKT	Patzer et al. [12]	US pediatric kidney failure	RR 0.48	Pediatric + preemptive + living donor
Waitlist activation	Talamantes et al. [13]	Community linguistic isolation	SHR 0.71	Community exposure, not individual proficiency
Living donor access	Mupfudze et al. [14]	OPTN + individual SDOH	13% vs 21% 3-y LDKT; income sHR 2.52	Observational SDOH association
Kidney geography	Arce et al. [15]	USRDS dialysis cohort	Marked attenuation after geographic/OPO adjustment	Historical era
Waitlist inactivity	Kulkarni et al. [16]	Post-KAS candidates	White vs Hispanic HR ~1.20	Inactivity resolution, not a sensitized-candidate effect
Contemporary DDKT	Madhusoodanan et al. [17]	290,763 candidates, 2015-2023	aHR 0.73 (95% CI 0.72-0.74)	DDKT after listing, not "any transplant"
Liver waitlist	Goyes et al. [18]	UNOS Share 35 era	SHR 1.23	Candidate waitlist outcome
NASH liver waitlist	Lim et al. [19]	18,562 NASH candidates	SHR 0.793 transplant; 1.173 mortality	Disease-specific
Liver geography	Volk et al. [20]	SRTR 2003-2008	HR 0.80 before geographic adjustment	Historical; observed disparity attenuated to non-significance after geographic adjustment
Donation approach	O'Connor et al. [21]	4,528 pediatric potential donors	OR 0.69	Pediatric; approach, not willingness
Culturally concordant LDKT	Gordon et al. [22]	HKTP pre/post	70% ratio increase	Program evaluation, not randomized
Post-LT liver outcomes	Thuluvath et al. [6]	National recipients	aHR 0.88 mortality; 0.90 graft failure	Association, not mechanism

Kidney transplantation: disparities across the care continuum

Pretransplant Care and Evaluation

Disparities begin before formal transplant evaluation. In a national USRDS analysis, Hispanic patients had 39% lower adjusted odds of receiving at least 12 months of predialysis nephrology care than White patients by 2014-2015 (adjusted odds ratio [OR]=0.61; 95% confidence interval [CI], 0.60-0.63) [11]. The study's cohort of more than one million adults represents the incident (new) end-stage kidney disease (ESKD) population who initiated maintenance dialysis, summed across the full 11-year study period from 2005 through 2015 rather than a single year. This cumulative figure should not be confused with more recent single-year USRDS new-ESKD incidence counts, which are considerably smaller [23]. In a cross-sectional survey of 167 hemodialysis patients, only 53% reported having completed a transplant evaluation, while provider non-referral and insufficient transplant knowledge were prominent barriers [24]. As the latter study did not show uniformly lower evaluation among Hispanic participants, it is best interpreted as evidence of modifiable referral and education barriers rather than a Hispanic-specific effect.

Living Donation and Socioeconomic Factors

Living donor access is strongly patterned by socioeconomic conditions. Mupfudze et al. found a three-year cumulative incidence of living-donor kidney transplantation (LDKT) of 13% among Hispanic candidates compared with 21% among non-Hispanic White candidates, with substantially greater LDKT likelihood at higher income levels [14]. In pediatric transplantation, Patzer et al. found that Hispanic children were 52% less likely to receive a preemptive LDKT (risk ratio [RR]=0.48; 95% CI, 0.35-0.67) [12] than White children; this estimate should not be generalized beyond pediatric preemptive living donation.

Waitlist Activation and Language Access

Talamantes et al. found that residence in a community in which at least 20% of households were linguistically isolated was associated with a 29% lower probability of achieving active waitlist status (subdistribution hazard ratio [SHR]=0.71; 95% CI, 0.67-0.76) [13]. As the exposure was community-level, the finding does not establish individual limited English proficiency as the cause. Medical distrust may also be relevant: Thompson et al. found greater distrust among non-White candidates and an association between values of distrust and lower activation among non-White candidates [25]. However, the activation effect was not estimated exclusively within Hispanic candidates.

Deceased-Donor Access, Geography, and Allocation Policy

Geography contributes importantly to deceased-donor kidney transplantation (DDKT). Arce et al. found lower DDKT access among Hispanic dialysis patients, with marked attenuation after adjustment for blood type, geography, and organ procurement organization factors [15]. Implementation of the Kidney Allocation System (KAS) in December 2024 was associated with increased transplantation rates among Hispanic candidates and an early narrowing of Hispanic-White differences [26]. However, disparities in preemptive transplantation specifically persisted and slightly worsened for Hispanic and Black candidates in the years after KAS implementation [27]. Kulkarni et al. found that White candidates were more likely than Hispanic candidates to resolve inactive waitlist status and return to active status (hazard ratio [HR] approximately 1.20) [16]. Additional studies identify inequity in repeat transplantation and around the estimated glomerular filtration rate (eGFR) threshold used for waitlist eligibility [28,29]. In a contemporary national analysis of 290,763 candidates from 2015 through 2023, Latino candidates had lower adjusted access to DDKT than White non-Latino candidates (adjusted HR=0.73; 95% CI, 0.72-0.74) [17]. A related national analysis of 98,863 adults preemptively waitlisted from 2009 through 2020 and followed through 2024 found a similar pattern. In the 2018-2020 era, Hispanic candidates were less likely than White candidates to receive a preemptive living-donor kidney transplant (adjusted SHR=0.61; 95% CI, 0.56-0.66) and were more likely to initiate dialysis before transplantation [30].

Liver transplantation: disparities across the care continuum

Referral and Evaluation

In a national study of 717,580 hospitalizations for decompensated cirrhosis from 2009 through 2018, Hispanic patients remained less likely than non-Hispanic White patients to receive liver transplantation by the end of the study period (OR=0.74; 95% CI, 0.70-0.78) [31]. A contemporary single-center cohort of 2,877 patients referred for liver transplantation found similar overall listing rates between Hispanic and non-Hispanic White patients (63% vs 64%) despite more frequently reported financial barriers among Hispanic patients, illustrating that aggregate listing rates alone can understate access barriers occurring earlier in the referral and evaluation process [32].

Waitlist Outcomes and Disease-Specific Disparities

In a national United Network for Organ Sharing (UNOS) analysis after Share 35, Hispanic candidates had a 23% higher adjusted competing risk of removal for death or clinical deterioration (SHR=1.23; 95% CI, 1.13-1.34) [18]. In nonalcoholic steatohepatitis (NASH) cirrhosis, Hispanic candidates had a lower probability of transplantation (SHR=0.793; 95% CI, 0.747-0.842) and higher waitlist mortality (SHR=1.173; 95% CI, 1.052-1.308) [19].

Geographic Variation and Allocation Policy

Geographic variation is a major system-level contributor. Volk et al. reported lower transplantation among Hispanic than White candidates in 2003-2008 (HR=0.80; 95% CI, 0.77-0.83), with the observed disparity attenuated to non-significance after adjustment for unequal regional distribution and organ availability [20]. Allocation policy can also modify disparity. Mazur et al. found improved transplantation likelihood and lower waitlist removal among Hispanic HCC candidates after exception-policy changes [33].

Center-Level Heterogeneity

National disparities are not reproduced uniformly at every center. Tincopa et al. found no significant ethnic difference in overall liver transplantation (46.9% vs 46.1%) or overall waitlist removal (35.0% vs 36.5%) among 375 adults at a center with a higher number of Hispanic patients [34]. This demonstrates meaningful center-level heterogeneity without proving that any single center characteristic caused the observed inequity.

Donor-Side Disparities

In an adjusted analysis of 4,528 pediatric potential organ donors, next-of-kin for Hispanic patients had 31% lower odds of being approached for donation than non-Hispanic White families (OR=0.69; 95% CI, 0.54-0.89) [21]. This identifies family approach as a potentially modifiable step. However, as the study was focused on pediatric patients, it should not be generalized to all organ procurement encounters, and a lower approach should not be equated with lower willingness to donate.

Donation disparities should therefore be broken down into into identification, referral, family approach, informed discussion, authorization or consent, and organ recovery. Interventions aimed at family attitudes cannot correct inequity that originates earlier through inconsistent institutional referral or approach.

Interventions to reduce transplant disparities

After implementation of the Hispanic Kidney Transplant Program (HKTP), Gordon et al. reported a 70% increase in the Hispanic-to-non-Hispanic White living-donor kidney transplantation (LDKT) ratio and a 91% increase in Hispanic waitlist additions. However, comparable high-volume centers in regions with a large Hispanic population did not show a similar increase [22]. A subsequent multicenter effectiveness-implementation study found benefit at the intervention site with greater implementation fidelity but not at the lower-fidelity site [35], suggesting that implementation quality may determine whether culturally targeted programs change outcomes.

Pande et al. described a dedicated Spanish-speaking kidney transplant program organized around language-concordant scheduling, education, navigation, and clinical communication [36]. A similarly designed Latino-focused clinic in North Carolina was associated with a 125% increase in Hispanic/Latino referrals, a 142% increase in waitlist additions, and a 145% increase in transplants within one year of implementation [37]. A bilingual telenovela-style intervention among 428 Hispanic patients with end-stage kidney disease improved transplantation-related knowledge [38]. Educational interventions should complement rather than substitute for structural changes in referral, evaluation, and access.

In a two-era liver study, Patnaik et al. reported substantially greater living donor liver transplantation likelihood and lower risk of waitlist death or clinical deterioration among Hispanic candidates in the later program era [39]. As multiple aspects of care can change between eras, the study supports living-donor infrastructure as a promising strategy without isolating a single causal program effect.

Racial disparities in living-donor liver transplantation are not specific to Hispanic patients. National pediatric waitlist data show that African American children are roughly half as likely to receive a living-donor liver transplant as White children. On the other hand, Asian children have utilization rates similar to or exceeding White children [40]. Living liver donors nationally remain predominantly White, with Black and Asian individuals together comprising fewer than 10% of all living liver donors over the past two decades [41]. American Indian/Alaska Native donors are too few to be reported separately in most national registries, reflecting a broader gap in disaggregated donor data for this population.

Post-transplant outcomes and the Hispanic transplant paradox

An important counterpoint to reduced access is the observation that Hispanic patients who ultimately undergo transplantation frequently experience outcomes comparable with or better than non-Hispanic White recipients. Here, the term Hispanic transplant paradox is used descriptively to denote the coexistence of reduced access with favorable outcomes among recipients; it does not imply a single underlying biological, behavioral, or selection mechanism [9].

In liver transplantation, Thuluvath et al. reported lower adjusted hazards of patient mortality (adjusted HR [aHR]=0.88; 95% CI, 0.84-0.92) and graft failure (aHR=0.90; 95% CI, 0.86-0.94) among Hispanic recipients [6]. Similarly, Althoff et al. reported favorable short- and long-term outcomes [7]. As these national registry analyses cover overlapping calendar periods, they are best treated as convergent rather than wholly independent evidence. Contemporary kidney data shows a similar pattern. Giusti et al. found lower rates of death and of the composite of death or kidney allograft loss among Hispanic recipients compared with non-Hispanic White recipients [8].

The mechanism remains uncertain. Selection into transplantation may contribute because patients who successfully navigate referral, evaluation, financial requirements, waitlisting, geography, and organ availability may differ systematically from those who do not reach transplantation. Existing studies do not establish selection as the predominant explanation. The principal equity implication is that available evidence does not support reduced access on the basis of systematically poorer expected post-transplant outcomes.

Discussion

Multi-Stage Attrition Across the Transplantation Pathway

The most important synthesis is not a single disparity estimate but the recurrence of disadvantage at multiple transitions. Kidney inequity can arise before listing, during evaluation and inactive status, and after active listing. Liver transplantation shows a similar sequence across hospitalization, referral, evaluation, waitlist survival, disease-specific pathways, and allocation. The same patients are not necessarily disadvantaged at every stage, and center-level null findings demonstrate meaningful heterogeneity.

Allocation reform can improve equity among active candidates without correcting disparities arising earlier through delayed specialty care, incomplete evaluation, financial barriers, language discordance, or limited living-donor opportunities. Equity strategies therefore need to address the full continuum rather than a single terminal decision.

Geography and Policy Responsiveness

Geographic adjustment markedly attenuated Hispanic-White differences in both kidney and liver transplantation in influential historical analyses [15,20]. Similarly, policy studies show that implementation of the Kidney Allocation System (KAS) in December 2014, and later HCC exception-policy changes, altered racial and ethnic differences in access [26,33]. These findings argue against treating disparity as an immutable characteristic of patients and caution against assuming that improvement in one era represents permanent resolution; contemporary post-KAS data continue to demonstrate residual DDKT disparity [17].

Modifiable Institutional Practices

Culturally and linguistically concordant kidney programs have been associated with increased waitlist additions and living-donor transplantation, while dedicated Spanish-speaking services and culturally tailored education address communication and navigation barriers [22,35,36,38]. Donor-side data identify family approach as a measurable institutional process that can be audited separately from consent or willingness [21].

Transplant centers and organ procurement organizations can directly measure referral, evaluation completion, active-status conversion, time inactive, living-donor progression, family approach, consent, transplantation, and waitlist outcomes by ethnicity and preferred language.

The Access-Outcome Paradox

The coexistence of reduced access with favorable post-transplant outcomes is the fourth major synthesis. The mechanism remains uncertain and should not be reduced to survivor-selection bias. Selection into transplantation is one potential contributor among multiple parameters such as patient-, social-, donor-, center-, socioeconomic-, behavioral-, and biological factors. The clinically important point is that lower access is not accompanied by evidence of systematically poorer transplant benefit among Hispanic recipients.

Implications for Transplant Equity

Hispanic transplant inequity is best understood as a systems problem involving interacting patient-, center-, regional-, and policy-level factors. Potential responses include earlier referral, professional language services and bilingual navigation, culturally concordant education, financial and logistical support for candidates and living donors, systematic review of waitlist inactivity, equitable family-approach practices, development of living-donor infrastructure, and continued equity evaluation during allocation-policy change. Representative barriers, supporting evidence, and potential system-level responses are summarized in Table 2. 

**Table 2:** Structural barriers, representative evidence, and potential system-level responses. Structural barriers identified across the transplantation care continuum, representative supporting evidence, potential system-level responses, and key limitations of the underlying evidence. DDKT: deceased-donor kidney transplantation; HCC: hepatocellular carcinoma; LDKT: living-donor kidney transplantation; LDLT: living-donor liver transplantation; LT: liver transplantation; SDOH: social determinants of health.

Stage	Barrier/finding	Representative evidence	Potential response	Evidence limitation
Pre-transplant kidney care	Reduced predialysis nephrology care	Purnell et al., 2020 [11]	Earlier referral; education triggers	Registry association
Evaluation	Linguistic isolation	Talamantes et al., 2017 [13]	Interpretation; bilingual navigation	Community-level measure
Evaluation	Medical distrust	Thompson et al., 2024 [25]	Continuity; culturally responsive communication	Not a Hispanic-specific activation estimate
Living donation	Socioeconomic gradient	Mupfudze et al., 2024 [14]	Donor financial/logistical support	Residual confounding
Waitlist inactivity	Lower reactivation	Kulkarni et al., 2019 [16]	Standardized inactivity review	Mechanism not established
DDKT geography	Regional access variation	Arce et al., 2013 [15]	Geographic equity monitoring	Historical era
Kidney post-listing	Residual contemporary disparity	Madhusoodanan et al., 2025 [17]	Ongoing center/region surveillance	Registry causality limits
Liver waitlist	Higher death/deterioration removal	Goyes et al., 2021 [18]	Earlier referral; center monitoring	Era/indication dependent
Metabolic liver disease	Lower LT/higher mortality	Lim et al., 2023 [19]	Targeted referral; SDOH support	Disease-specific cohort
HCC allocation	Policy responsiveness	Mazur et al., 2024 [33]	Equity analysis during policy change	Era comparison
Center-level liver care	National disparity not uniform	Tincopa et al., 2025 [34]	Study high-performing processes	Single center
Organ donation	Lower family approach	O'Connor et al., 2025 [21]	Standardize approach; audit rates	Pediatric
Culturally concordant care	LDKT access improvement	Gordon et al., 2015 [22]	Language/culture-concordant programs	Pre-post
Implementation fidelity	Benefit varied by fidelity	Gordon et al., 2022 [35]	Measure fidelity	Few centers
Education	Knowledge improvement	Forster et al., 2016 [38]	Tailored multimedia + navigation	Intermediate endpoint
Living donor liver	Later-era improvement	Patnaik et al., 2026 [39]	LDLT infrastructure	Era comparison

These strategies are targets for implementation and prospective evaluation rather than formally graded clinical recommendations because the supporting evidence is predominantly observational and varies in design, population, and endpoint.

The interaction of these barriers across the transplantation care continuum and representative evidence supporting each stage are summarized in Figure 2.

**Figure 2:**
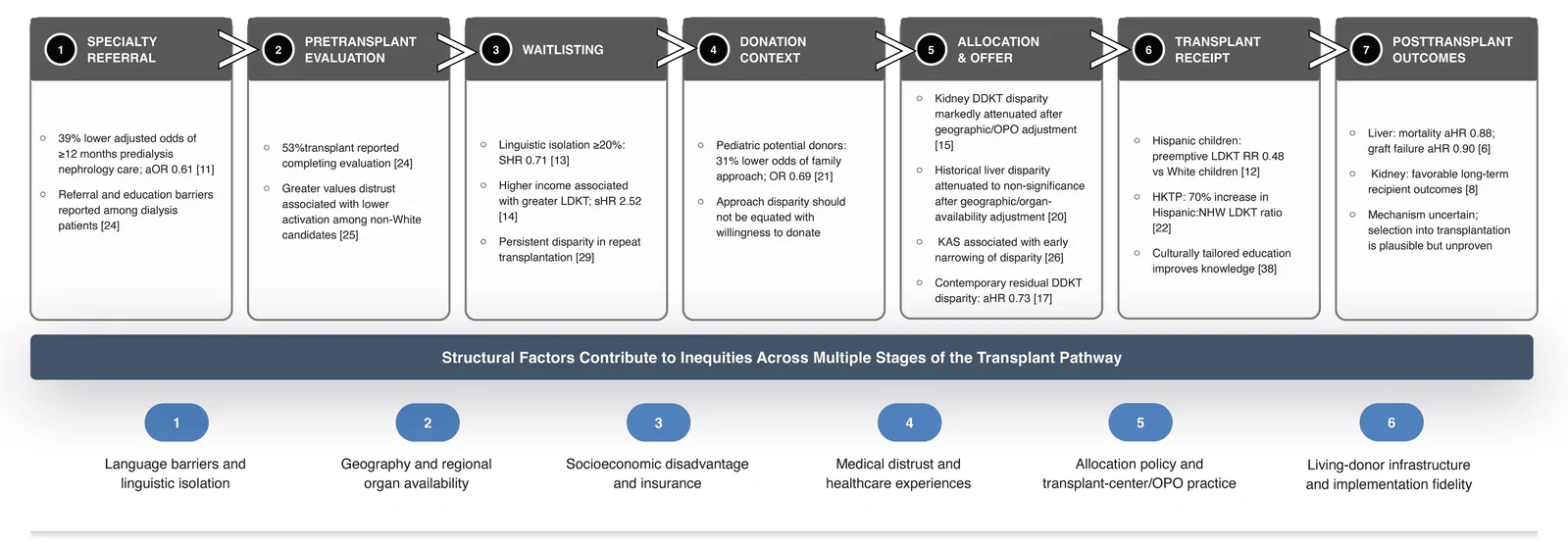
Structural barriers and modifiable processes across the transplantation care continuum. Representative evidence illustrates how inequities affecting Hispanic patients may arise across specialty referral, transplant evaluation, waitlisting, donation, organ allocation, transplant receipt, and posttransplant outcomes. Effect estimates are study-specific and should not be directly compared across stages. Structural drivers may interact across levels; the diagram is intended as a conceptual synthesis rather than a causal model. DDKT: deceased-donor kidney transplantation; HKTP: Hispanic Kidney Transplant Program; KAS: Kidney Allocation System; LDKT: living-donor kidney transplantation; OPO: organ procurement organization; OR: odds ratio; RR: risk ratio; SHR: subdistribution hazard ratio; aHR: adjusted hazard ratio; aOR: adjusted odds ratio.

Strengths and limitations

This review integrates evidence across kidney and liver transplantation, multiple stages of the care continuum, and diverse study designs using a structured, multi-database search. Its principal strengths are the stage-specific synthesis, explicit treatment of overlapping registry cohorts as convergent rather than independent evidence, and attention to policy era and study context. Hispanic patients account for a comparatively smaller share of the donor pool (16.3% of living kidney donors and 14.6% of deceased liver donors) than of the candidate population [5]. However, this population-level figure does not capture stage-specific donor identification, approach, or consent processes, which are examined separately in this review.

The evidence base is predominantly observational, spans evolving allocation and clinical practices, and incompletely captures heterogeneity within Hispanic populations. Consistent with the narrative design, formal risk-of-bias scoring and duplicate screening or adjudication were not performed. Intervention studies remain relatively limited and largely single-center or nonrandomized, underscoring the need for larger multicenter prospective evaluations of durable strategies to improve transplant equity.

## Conclusions

Transplantation inequities affecting Hispanic patients in the United States arise across multiple, potentially modifiable stages of the care continuum, supporting coordinated interventions across referral, evaluation, waitlisting, allocation, and donation. As Hispanic recipients generally achieve post-transplant outcomes comparable with or better than non-Hispanic White recipients, improving equity should focus primarily on reducing barriers to transplant access.
